# DPA shows comparable chemotherapy sensitizing effects as EPA upon cellular incorporation in tumor cells

**DOI:** 10.18632/oncotarget.27236

**Published:** 2019-10-15

**Authors:** Francina J. Dijk, Miriam van Dijk, Bram Dorresteijn, Klaske van Norren

**Affiliations:** ^1^Danone Nutricia Research, Nutricia Advanced Medical Nutrition, Utrecht, The Netherlands; ^2^Nutritional Biology, Department of Human Nutrition and Health, Wageningen University, Wageningen, The Netherlands

**Keywords:** DPA, fish oil, nutrition, chemotherapy, cancer

## Abstract

Dietary supplementation with ω-3 polyunsaturated fatty acids (PUFAs) has been reported to enhance the sensitivity of tumor cells towards chemotherapy. Most enhancing effects are described for ω-3 PUFAs EPA and DHA; less evidence is available with the intermediate DPA. We studied the chemotherapy enhancing effects of EPA, DPA and DHA in murine colon C26 adenocarcinoma cells and showed that DPA displayed similar chemosensitizing effects as EPA. Moreover, EPA supplementation increased cellular DPA content. In a C26 tumor-bearing mouse model, we studied the incorporation of ω-3 PUFA in tumor and skeletal muscle after a diet with different ω-3 PUFA sources. Although little DPA was present in the fatty acid food sources, in those that contained considerable EPA concentrations, DPA levels were higher in tumor and muscle tissue. From these studies, we conclude that EPA and DPA show chemosensitizing effects and that intake of EPA or EPA-containing nutrition leads to increased cellular DPA content by elongation. These findings support the use of ω-3 PUFA containing nutritional supplementations in cancer patients during chemotherapy treatment.

## INTRODUCTION

Cancer, a major disease worldwide, is often accompanied by malnutrition and the involuntary loss of muscle and fat mass that cannot be restored with normal food intake (i.e. cachexia) [1, 2]. Although cancer treatment therapies are continuously improving, they often do not specifically target cancer cells, and lead to toxicity and cell death of normal cells [1]. Specialized nutritional care might improve clinical outcomes and nutritional status of cancer patients by protecting normal cells and enhancing the efficacy of treatment [1, 3–5]. Recently, the ESPEN guidelines on nutrition in cancer patients have been published, showing new consensus on nutritional needs of cancer patients [1]. One of the recommendations in the ESPEN guidelines is the use of ω-3 polyunsaturated fatty acids (PUFAs) or fish oil, for patients with advanced cancer undergoing chemotherapy and at risk of weight loss or malnutrition. Clinical studies showed lower systemic inflammation and improvements in appetite, energy intake, body weight and lean body mass which resulted in an improved quality of life with (fish oil-derived) ω-3 PUFA supplementation [4, 6–8]. ω-3 PUFAs are rapidly incorporated into cell membrane phospholipids [9], display anti-inflammatory effects and have been found to enhance sensitivity to chemotherapy [4, 6, 10–14]. Still, further research is required to determine the exact mechanisms behind the effects of ω-3 PUFAs.

Most effects of ω-3 PUFAs are ascribed to eicosapentaenoic acid (EPA, 20:5 ω-3) and docosahexaenoic acid (DHA, 22:6 ω-3). Docosapentaenoic acid (DPA, 22:5 ω-3) is the intermediate ω-3 PUFA between EPA and DHA. DPA is also present in fish oil, but in relatively small amounts [15], and is therefore less well studied. However, it has been reported that DPA incorporation reduces platelet aggregation, improves lipid metabolism, and reduces inflammation in several cell and animal models (reviewed in [15–17]). In addition, DPA showed anti-proliferative and pro-apoptotic effects in colorectal carcinoma cells [18]. Some authors suggest that DPA is the storage depot for EPA and DHA in the human body [19]. DPA can be synthesized by the elongation of EPA in cells, which is mediated by the enzymes elongase-2 (ELOVL2) and elongase-5 (ELOVL5) [17, 20]. DPA can also be converted to DHA, although several studies showed that this occurs seldom and seems to be limited to the liver [21–23]. Retro-conversion of DPA to EPA or from DHA to DPA can also occur with involvement of the enzymes peroxisomal acyl-CoA oxidase and one cycle of β-oxidation [17]. To our knowledge, DPA has not been studied regarding its effect on chemotherapy sensitivity during cancer or incorporation in tumor cells. Since EPA and DHA have been shown to enhance chemotherapy sensitivity, we assume that DPA might also display similar properties. In this study, we investigated the effects of EPA, DHA and DPA on cell viability and caspase 3/7 activity in murine colon adenocarcinoma (C26) cells treated with the chemotherapeutics doxorubicin and cisplatin. In addition, we measured the fatty acid composition of the cells after incubation with EPA, DPA and DHA. Furthermore, we used the C26 tumor mouse model of cancer cachexia to study the effects of ω-3 PUFAs on cachexia parameters, immune function and the incorporation of ω-3 PUFAs into tumor and muscle phospholipids after nutritional intervention with different ω-3 PUFA sources, containing different ratios of EPA, DPA and DHA (i.e. fish oil, pure EPA and tuna oil). We expect that the incorporation of the ω-3 PUFAs may play a crucial role in its efficacy and, therefore, we determined ω-3 fatty acid composition in the cells and tissues after supplementation.

## RESULTS

### C26 adenocarcinoma cells

#### Cell viability and apoptosis of C26 adenocarcinoma cells

**Figure 1:**
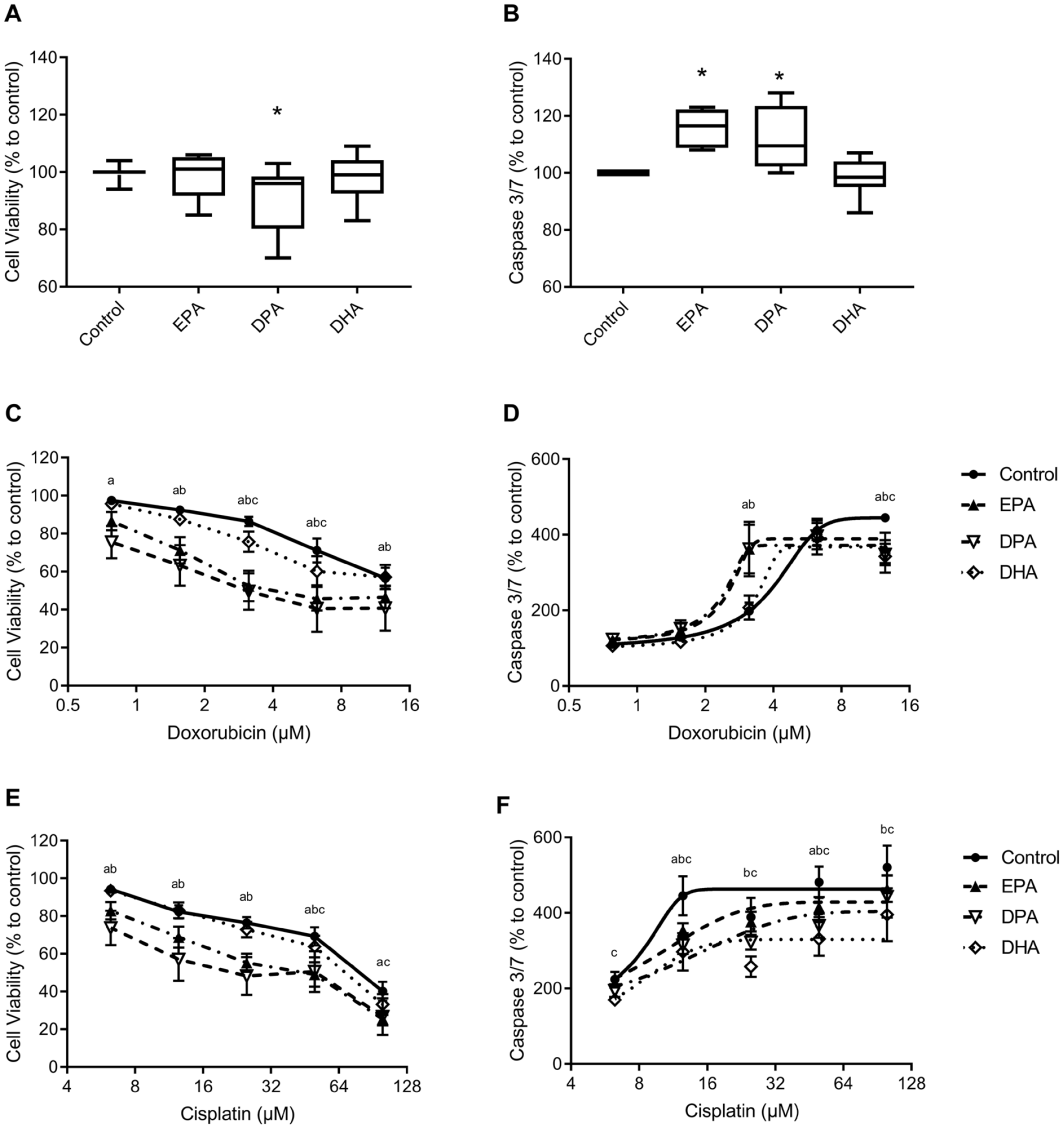
Cell viability and caspase 3/7 activity of C26 adenocarcinoma cells. C26 cells were pre-incubated with EPA, DPA, DHA or solvent control for 4 days (**A, B**) and thereafter incubated with doxorubicin (**C, D**) or cisplatin (**E, F**) for 24 h. Values are mean ± SEM of 3–7 individual experiments. ^*^Significantly different (*P* & 0.05) from control; significantly difference between (*P* & 0.05) control and a) EPA, b) DPA and c) DHA.

Figure 1A shows the effect of EPA, DPA and DHA on C26 cell viability without chemotherapy. A significant decrease in cell viability was measured after DPA treatment (*P* = 0.007), while EPA and DHA had no effect on cell viability. Both EPA and DPA showed significantly higher caspase 3/7 activity (*P* = 0.001 for EPA and *P* = 0.01 for DPA) compared to control cells treated with solvent only (Figure 1B) while DHA showed no effect.

With increasing doxorubicin (DOX) concentration, without PUFAs, the cell viability decreased while caspase 3/7 activity increased significantly compared to control cells (DOX > 1.56 µM, *P* & 0.006, Figure 1C, 1D). Pre-incubation with 50 µM EPA significantly strengthened the effect of DOX on cell viability at all concentrations (*P* & 0.05) and with 50 µM DPA at DOX concentrations higher than 0.78 µM (*P* & 0.05). After preincubation with 50 µM DHA, the cell viability decreased at DOX concentrations 3.13 and 6.25 µM (*P* & 0.001 compared to control cells at same DOX concentration, Figure 1C). IC_50_ values for cell viability of DOX are lower with EPA and DPA (6.24 and 4.16, respectively) compared to control and DHA (15.4 and 14.25, respectively), as shown in Table 1. Caspase 3/7 activity was significantly enhanced with EPA and DPA at 3.13 µM DOX (*P* & 0.0001), although at 12.5 µM DOX significantly lower caspase 3/7 activity was measured with EPA, DPA and DHA compared to control (*P* & 0.02, Figure 1D).

**Table 1:** IC_50_ values of cell viability (WST) for C26 cells treated with DOX or CIS with or without ω-3 PUFAs

	**DOX**	**CIS**
Control	15.38 ± 1.45	80.66 ± 1.48
EPA	6.24 ± 2.51	35.50 ± 1.91
DPA	4.16 ± 3.85	27.41 ± 3.84
DHA	14.25 ± 2.04	63.89 ± 1.77

Data represent mean IC_50_ in µM ± SEM.

Treatment of C26 cells with cisplatin (CIS), without PUFAs, showed a dose-dependent decrease in cell viability (CIS > 6.25 µM, *P* & 0.0001 compared to control) with a significant increase in caspase 3/7 activity which plateaued at 12.5 µM CIS (*P* & 0.002 for all concentrations compared to control). Preincubation with EPA significantly decreased cell viability of CIS at all concentrations (*P* & 0.002) and DPA at concentrations 6.25, 12.5, 25, 50 µM CIS (*P* & 0.04, Figure 1E), DHA lowered cell viability at 50 µM CIS (*P* = 0.045) and 100 µM CIS (*P* = 0.011) compared to control. IC_50_ values for cell viability showed lower values with CIS and EPA or DPA supplementation compared to control (Table 1). Caspase 3/7 activity was significantly lower after treatment with EPA at 12.5 and 50 µM CIS (*P* = 0.011 and *P* = 0.034, respectively), with DPA at 12.5 until 100 µM CIS (*P* & 0.017) and with DHA at all CIS concentrations compared to control cells (*P* & 0.034, Figure 1F).

#### Fatty acid composition of culture medium and total cell lysate of C26 adenocarcinoma cells

**Figure 2:**
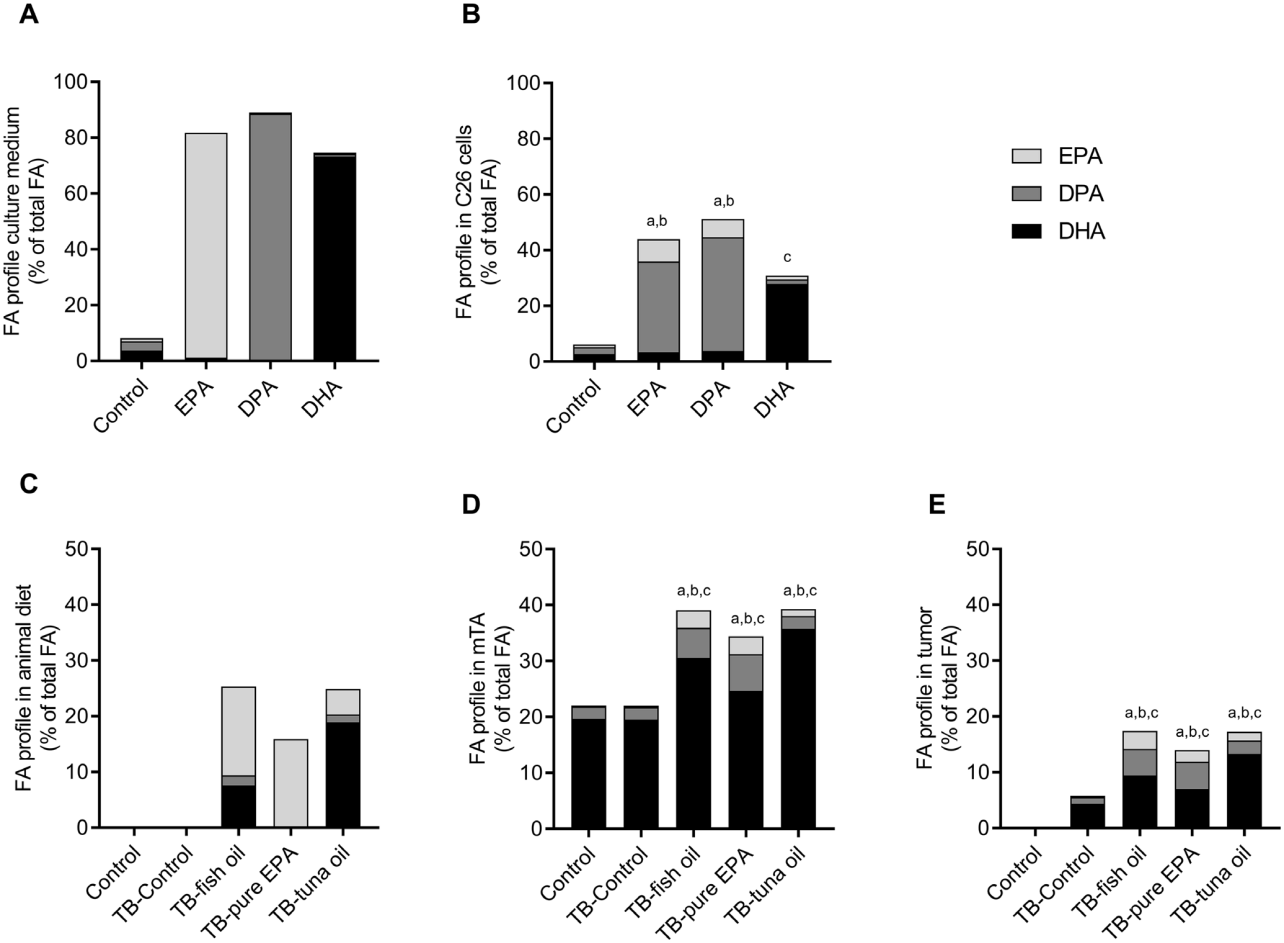
ω-3 PUFA compositions. Fatty acid composition of C26 culture medium (**A**) and C26 cell lysate after 4 days treatment with 50 µM EPA, DPA, DHA or solvent control (**B**). Fatty acid composition of the diets in the C26 mouse model (**C**) and of mTA muscle (**D**) and tumor (**E**) in mice. Values represent mean of at least 4 independent experiments in C26 cells, *n* = 9–10 for the C26 mouse model. Significant difference (*P* & 0.05) between control and intervention of EPA (a), DPA (b) and DHA (c).

Figure 2A shows the ω-3 fatty acid composition of the culture medium supplemented with control, EPA, DPA or DHA. Figure 2B shows the ω-3 fatty acid composition of the total cell lysate. EPA supplementation led to a significant higher content of both EPA (8.1%, *P* & 0.0001) and DPA (32.6%, *P* & 0.0001) in the cellular lysate compared to control. DPA supplementation led to a significant increase of EPA (6.6%, *P* & 0.0001) and DPA (40.8%, *P* & 0.0001). Supplementation with DHA increased cellular DHA content significantly in the cell lysate (28%, *P* & 0.0001).

### C26 tumor-bearing mouse model

#### Body weight and composition

Table 2 shows the composition of the different diets the tumor-bearing mice received. At day 20 after tumor inoculation, mice were sacrificed and body weight, body composition, muscle and organ weights and immune function (contact hyper sensitivity (CHS) measurement) were measured and summarized in Table 3. Food intake was similar in all groups. Significant increases in the control (C) group compared to the TB-C group and were found in carcass weight, fat mass (total and epididymis fat), bone mineral density (BMD) and bone mineral content (BMC), and hindlimb muscles tibialis anterior (mTA), soleus (mS), gastrocnemius (mG) (*P* & 0.013). In C, thymus weight was significantly higher (*P* & 0.0001), whereas spleen (*P* = 0.0001) and liver (*P* = 0.045) were significantly lower compared to TB-C. No significant differences were observed between body weight, body composition, skeletal muscle weights or tumor weight between tumor-bearing control and tumor-bearing experimental diet groups. In the tumor-bearing fish oil (TB-FO) group, heart weight was significantly lower (*P* = 0.023) and thymus weight was significantly higher (*P* = 0.007) compared to TB-C. Both the tumor-bearing EPA (TB-EPA) and tumor-bearing tuna oil (TB-TO) group showed significantly lower spleen and liver weight (*P* & 0.04) compared to TB-C. CHS showed higher levels in the control group compared to TB-C (*P* & 0.005). No differences were observed in CHS in the intervention groups compared to TB-C.

**Table 2:** Nutritional composition of control and experimental diets used in the C26 tumor mouse model

Ingredients	g/kg dry matter	C	TB-C	TB-FO	TB-EPA	TB-TO
Carbohydrates	Cornstarch	466.0	466.0	466.0	466.0	466.0
	Dex. Cornstarch	155.0	155.0	155.0	155.0	155.0
	Sucrose	100.0	100.0	100.0	100.0	100.0
Fibers	Cellulose	50.0	50.0	50.0	50.0	50.0
Protein	Casein	140.0	140.0	140.0	140.0	140.0
Fat	Soy bean oil	40.0	40.0	17.90	33.07	11.96
	Fish oil	-	-	22.10	-	-
	Pure EPA	-	-	-	6.93	-
	Tuna oil	-	-	-	-	28.04
% ω-3	EPA C20:5 ω-3	-	-	15.9%	15.9%	4.6%
	DPA C22:6 ω-3	-	-	1.8%	-	1.4%
	DHA C22:5 ω-3	-	-	7.6%	-	18.9%
Others	Mineral mix	35.0	35.0	35.0	35.0	35.0
	Vitamin mix	10.0	10.0	10.0	10.0	10.0
	Choline Bitrate	2.5	2.5	2.5	2.5	2.5
	*tert*-butylhydroquinone	0.008	0.008	0.008	0.008	0.008
Total kcal		3601	3601	3601	3601	3601

Diets are based on AIN93-M with soy bean oil in the control and tumor-bearing control group partly replaced by fish oil in the TB-FO group, pure EPA in the TB-EPA group or tuna oil in the TB-TO group. All diets were equal in amount of protein, carbohydrates, fibers, fat, mineral and vitamin mix and kcal per kg dry matter.

**Table 3:** *In vivo* characteristics of mice after supplementation with control or experimental diet, measured at day 20

Parameters	C	TB-C	TB-FO	TB-EPA	TB-TO
n	10	9	10	10	10
Body Weight (g)	23.5 ± 0.6	21.8 ± 0.7	23.0 ± 0.8	21.4 ± 0.5	21.9 ± 0.8
∆Body Weight d20-d0	1.1 ± 0.3	–0.5 ± 0.6	0.8 ± 0.6	–0.9 ± 0.6	–0.4 ± 0.8
Carcass Weight (g)	23.5 ± 0.6^*^	19.5 ± 0.7	20.9 ± 0.8	19.3 ± 0.5	19.7 ± 0.8
Tumor Weight (g)	-	2.2 ± 0.2	2.1 ± 0.1	2.1 ± 0.1	2.2 ± 0.1
Tumor Volume (cm^3^)	-	1.7 ± 0.2	1.7 ± 0.2	1.8 ± 0.1	1.7 ± 0.3
Mean food intake (g)	4.0 ± 0.1	3.9 ± 0.1	3.8 ± 0.1	3.9 ± 0.1	3.9 ± 0.1
DEXA results					
LBM (g)	20.0 ± 0.5	19.7 ± 0.6	20.1 ± 0.6	18.9 ± 0.4	19.5 ± 0.6
Fat mass (g)	5.9 ± 0.3^*^	4.3 ± 0.2	4.8 ± 0.3	4.4 ± 0.2	4.5 ± 0.3
BMD (g/cm^2^)	0.052 ± 0.002^*^	0.048 ± 0.001	0.050 ± 0.001	0.049 ± 0.001	0.050 ± 0.001
BMC (g/cm)	0.47 ± 0.02^*^	0.42 ± 0.01	0.43 ± 0.01	0.44 ± 0.01	0.44 ± 0.02
Skeletal Muscle (mg)					
mTA	44.1 ± 0.9^*^	36.4 ± 1.5	38.8 ± 1.2	35.8 ± 1.1	35.7 ± 1.1
mEDL	9.8 ± 0.3	7.9 ± 0.3	8.3 ± 0.3	8.1 ± 0.4	8.0 ± 0.3
mSoleus	6.4 ± 0.2^*^	5.6 ± 0.3	5.9 ± 0.4	5.5 ± 0.2	5.4 ± 0.2
mGM	133.0 ± 4.1^*^	111.5 ± 4.4	121.2 ± 4.6	113.6 ± 2.8	109.2 ± 4.5
Organs (mg)					
Spleen	87.7 ± 1.6^*^	269.0 ± 17.1	231.1 ± 9.8	223.9 ± 15.0^*^	199.1 ± 19.0^*^
Kidney	403.7 ± 10.6	385.9 ± 14.6	388.5 ± 14.3	367.2 ± 15.0	373.5 ± 18.1
Liver	1114 ± 38^*^	1228 ± 59	1128 ± 39	1109 ± 28^*^	1066 ± 32^*^
Thymus	31.3 ± 2.7^*^	13.2 ± 1.8	21.1 ± 2.1^*^	15.1 ± 1.6	18.0 ± 2.0
Heart	145 ± 3.3	139 ± 5.4	125 ± 4.2^*^	130 ± 4.0	130 ± 3.9
Lungs	155 ± 3.6	166 ± 4.5	176 ± 8.1	176 ± 7.0	167 ± 5.6
Epididymal fat	219 ± 23.7^*^	85 ± 24.4	129 ± 25.6	85 ± 16.0	96 ± 25.2
CHS test (µm)	202.7 ± 11.9^*^	164.1 ± 12.9	158.7 ± 11.3	149.1 ± 6.4	154.9 ± 10.6

Data are mean ± SEM of the control (C) group, tumor-bearing control (TB-C) group, tumor-bearing groups after supplementation with fish oil (TB-FO), pure EPA (TB-EPA) or tuna oil (TB-TO). Calculation of Tumor Volume = 0.5 *×* (length × width^2^)/1000. ^*^Indicates significant difference (*P* & 0.05) from the TB-C group, measured with ANOVA and LSD post hoc analysis.

#### Phospholipid fatty acid composition of diet, mTA and tumor

Figure 2C shows the ω-3 phospholipid fatty acid composition of the control and intervention diets that were given to the mice. The phospholipid ω-3 fatty acid composition of the tibialis anterior muscle (mTA) and tumor at day 20 are shown in Figure 2D and Figure 2E respectively. In mTA, the percentage of EPA was significantly higher in all diet intervention groups (*P* & 0.002) compared to the TB-C group; the percentage DPA was significantly higher in the TB-FO and TB-EPA groups (*P* & 0.001). Remarkably, mTA contains about 20% DHA independent of the diet (as shown in the C and TB-C groups), although with the addition of supplementary DHA in the diet, the percentage DHA in mTA was significantly higher in all diet intervention groups (*P* & 0.003, Figure 2D) compared to TB-C. Furthermore, in mTA, the percentage of unsaturated fatty acid other than EPA, DPA and DHA, is lower in the intervention groups (*P* & 0.001) compared to TB-C (data not shown). In the tumor, the percentage of phospholipids EPA, DPA and DHA was significantly higher in all intervention groups compared to TB-C (*P* & 0.002). On the contrary, total unsaturated fatty acids, other than EPA, DPA and DHA, were significantly lower in the intervention groups (*P* & 0.001) compared to TB-C (data not shown).

## DISCUSSION

This study shows that EPA incorporation in cells leads to DPA by elongation and that both EPA and DPA supplementation have a significant beneficial effect on the sensitivity of chemotherapeutics doxorubicin and cisplatin. However, the mechanism behind the chemotherapy enhancing effects on both chemotherapeutics seems to be different. The effect of EPA and DPA on sensitivity to doxorubicin seems to be related to caspase 3/7 activated loss of cell viability. The effect of EPA and DPA on sensitivity to cisplatin seems to work independent of caspase 3/7 activation. Interestingly, the effects of EPA and DPA in chemotherapy treated cells were similar, and fatty acid analysis of cell lysates showed that most of the supplemented EPA was elongated to DPA. This phenomenon was also observed in the C26 tumor model where mice received a diet with fish oil or pure EPA. Here we observed a relative low amount of EPA incorporated in muscle and tumor tissue, while DPA content increased to much higher levels than in the diet.

Our data of EPA-induced sensitivity to chemotherapy are in line with literature, as reviewed by [4] and [24], however, our findings of DPA have not been described before. Enhanced caspase 3/7 activity with EPA and DHA have been described in many tumor cell experiments and animal experiments [2] (reviewed in [25–28]). We found only limited effects of DHA and could not confirm the effect of DHA on caspase 3/7 activity, which is in line with Jacobsen *et al.* [29] and Calder [13]. These authors suggested that the mechanism of DHA might work via ER stress and disturbed Ca^2+^ homeostasis and does not involve caspase activities. Calder [13] described how the effects of DHA can be explained by the induction of oxidative stress.

In contrast to EPA and DHA, less is known about the chemotherapy sensitivity of DPA. To our knowledge there is one study of Morin *et al.* [18] that showed anti-proliferative and pro-apoptotic effects of monoacylglyceride DPA (MAG-DPA) in HCT116 colorectal adenocarcinoma cells; however, not in combination with chemotherapy. The study also showed a significant decline in tumor growth following MAG-DPA supplementation in a HCT116 mouse xenograft model. Our *in vitro* study not only shows the ability of DPA to decrease viability and increase apoptosis, but also shows the ability of DPA to enhance the effect of chemotherapy treatment in tumor cells, which to our knowledge has not been described before. In contrast to Morin, our *in vivo* study did not show any effect on tumor growth with a DPA-containing fish oil diet.

As an addition to the *in vitro* approach, we also conducted an *in vivo* experiment using the C26 tumor mouse model in which mice received a normal diet or a diet containing different ω-3 PUFA sources. In alignment with other studies, we observed characteristics of cachexia, i.e. a decrease in carcass weight, fat mass (total and epididymal fat), BMD and BMC and hindlimb muscles mTA, mS and mG. Of note, when comparing our results to the study of Faber *et al.* [30] and Van Norren *et al.* [31], our observed cachexia characteristics were less severe (i.e. carcass weight, epididymal fat, muscle weights). No beneficial effects of improved cachexia characteristics or decreased tumor growth were measured in the intervention groups receiving the different ω-3 PUFA sources. However, we did see an increase in liver and spleen weight in the TB-C group compared to the control group, which is indicative for cachexia and might be due to increased protein synthesis in the liver by the production of acute phase proteins and increased fibrinogen production [32–34]. Supplementation with all types of ω-3 PUFA sources resulted in a normalization of liver weights and reduced spleen weights. A higher dosage of the ω-3 PUFA might have shown enhanced effects on cachexia characteristics, additional mouse experiments with different dosages of ω-3 PUFA would give more insight. Our results are in line with the study of Van Norren *et al.* [31] with the same mouse model showing that fish oil alone (with the same ω-3 PUFA concentration) did not improve cachexia symptoms. However, Van Norren *et al.* showed that a diet with fish oil in combination with high protein and leucine resulted in less severe cachexia outcomes. A multifactorial approach seems to be the favorable strategy to combat cancer cachexia, according to Argiles [35, 36], Murphy [37] and Bjorklund [38]. In our study, feeding of mice with a fish oil containing diet resulted in a shift in phospholipid composition of muscle and tumor towards ω-3 PUFA within 21 days. An interesting but unclarified finding is the high DHA content in mTA which is also present in the control and TB-C group.

Similar to our *in vitro* observations, we also observed a shift from EPA to DPA in tumor and muscle tissues in tumor-bearing mice. Observations of elongation from EPA to DPA are repeatedly reported in several cell types varying from endothelial cells [39], B Lymphomas [40] and preadipocytes [41] and many others (reviewed in [17]), accompanied by quite some beneficial effects of DPA. Yet, there is still a lot of speculation about the mechanism behind DPA and its elongation from EPA. The differences in molecular structure changes the membrane order and fluidity and might explain a preference for DPA incorporation [41, 42]. The suggestion that DPA serves as a reservoir [19] is also a plausible explanation, although our findings show less retro-conversion of DPA to EPA. All experiments show an increase in DPA content after consumption of EPA or DPA, but not with DHA.

A limitation of the present *in vitro* study is that we cannot discriminate between loss of cell viability (measured by WST) due to cell death or a decreased viability of existing cells. However, with an increase in caspase 3/7 activity (only with doxorubicin treatment) the assumption of cell death is plausible. Furthermore, this study is limited to the effects of doxorubicin and cisplatin on C26 adenocarcinoma cells. Both chemotherapeutics are indicated for a broad range of cancer types, although not specific for the treatment of colorectal cancer as suggested by the use of C26 adenocarcinoma cells. Chemotherapy treatment concentrations were chosen to observe concentration dependent decreases in C26 cell viability for both doxorubicin and cisplatin. In the clinic, human plasma levels of patients treated with doxorubicin, do not exceed 5 µM [19], which is in the range of the concentrations tested in our *in vitro* assay. Human plasma concentrations of cisplatin and hydrolyzed cisplatin are in the nanomolar range [43], while we tested much higher concentrations to be able to measure decreases in cell viability. In the C26 tumor mouse model, we only studied the effect of fish oil supplementation on tumor and cachexia characteristics, an interesting next step would be to study the effect of EPA and DPA containing supplements in a chemotherapy treated C26 tumor mouse model. Furthermore, we observed elongation of EPA to DPA in *in vitro* and *in vivo* animal experiments, but did not study the possible mechanism behind this phenomenon. Several possible mechanisms are discussed in literature, however the exact reason for elongation is still unclear. Further research is necessary to obtain more clarity on this phenomenon.

Our main finding is that EPA and DPA, and to a lesser extend DHA, show doxorubicin and cisplatin chemotherapy enhancing effects in C26 adenocarcinoma cells *in vitro* and also directly impair the tumor cells. These finding are supportive for the use of ω-3 PUFA containing nutritional supplements in cancer patients as such or when receiving chemotherapy treatment. Furthermore, we observed an unexpected shift from EPA to DPA by elongation in cellular content or phospholipid composition in the *in vitro* and *in vivo* experiments. Further exploration of this elongation could obtain more information in the mechanism and benefits of this phenomenon.

## MATERIALS AND METHODS

### *In vitro* experiments

#### Cell culture and incubations

Murine C26 adenocarcinoma cells (ATCC) were plated in 96-well plates (Costar) at 5.0 × 10^3^ cells/well in RPMI 1640 (Life Technologies), provided with 10% FBS (Fisher Scientific) and 1% penicillin-streptomycin. The next day, cells were supplemented with 50 μM EPA, DPA or DHA (Sigma Aldrich) or culture medium with an equal amount of solvent (0.01% ethanol + 2.5% essentially fatty acid free BSA, Sigma Aldrich) as control for 4 days. Thereafter, cells were washed to remove any remaining ω-3 fatty acids and a 24 h incubation with a concentration series of doxorubicin (DOX; 0.2% liquid, Pharmachemie) or cisplatin (CIS; 0.1% liquid Platosin, Pharmachemie) followed. After chemotherapy incubation, cell viability was measured by WST-1 (Roche Diagnostics) and apoptosis was measured by Caspase-Glo 3/7 Assay (Promega), both were used according to manufacturer’s guide. IC_50_ values (Table 1) represent the chemotherapy concentration that is required to obtain 50% inhibition of cell viability measured by WST-1. Calculation of IC_50_ values is based on the raw data of all experiments and are calculated using non-linear fit in GraphPad Prism 8.

#### Fatty acid analysis

For fatty acid uptake analysis, cells were plated in 6-well plates (Costar) in a concentration of 1.5 × 10^5^ cells/well and for 4 days supplemented with 50 μM EPA, DPA, DHA or maintained in a control condition which was given an equal volume of solvent. Then cells were washed and trypsinized. After detachment, cells were collected and centrifuged for 5 min at 1500 rpm. The supernatant was removed and cells were lysed in ice-cold demineralized water and vortexed. Fatty acids were analyzed by GC, as previous described by Faber *et al.* [30].

### C26 tumor-bearing mouse model

#### Animals and diets

All experimental procedures were approved by an external, independent Animal Experimental Committee (DEC consult, Bilthoven, the Netherlands) and complied with national legislation and the principles of good laboratory animal care following the European Directive for the protection of animal used for scientific purposes. Syngeneic male CD2F1 mice (BALB/c x DBA/2, Charles River, the Netherlands) were 6–7 weeks of age at the start of the experiment. Animals were housed individually in a climate-controlled animal care facility (12:12 dark-light cycle with a constant room temperature of 21 ± 2°C, humidity 55 ± 5%). All animals had free access to food and drinking water. Upon arrival, animals were allowed to acclimatize for 1 week and subsequently randomized into 5 groups of 10 mice based on their bodyweight (BW). One group served as a control group (C), receiving a control diet, a tumor-bearing control group (TB-C) receiving a control diet and three tumor-bearing experimental groups receiving different experimental diets. All diets were based on the AIN93-M (Research Diet Services, Wijk bij Duurstede, The Netherlands). The control diet contained per kg food: 140 g protein (100% casein), 721 g carbohydrates and 40 g fat (100% soy bean oil). The experimental diets were adapted by replacing part of the soy bean oil by 22.1 g fish oil (TB-FO), or 6.93 g pure-EPA (TB-EPA) or 28.04 g tuna oil (TB-TO). Specific diet composition of each group is shown in Table 2.

#### Experimental design

Murine C26 adenocarcinoma cells were used to induce cachexia in mice [44–46]. C26 cells were cultured *in vitro* with RPMI 1640 supplemented with 5% FBS and 1% penicillin-streptomycin. Tumor cells were trypsinized in a sub-confluent state and, after washing, suspended in Hanks’ balanced salt solution (HBSS) (Life Technologies) at a concentration of 2.5 × 10^6^ cells/ml. Tumor cells (5 × 10^5^ cells in 0.2 ml) were inoculated, under general anesthesia (isoflurane/N_2_O/O_2_), subcutaneous into the right inguinal flank of mice in the tumor-bearing groups. Mice in the control group received a sham injection with 0.2 ml HBSS. Body weight and food intake were measured three times a week. Tumor size (length and width) was measured two times to examine tumor development. To study effects on the immune system, a Contact Hyper Sensitivity test (CHS) against oxazolone was performed. Briefly, on day 8, all animals were sensitized with 150 µl 3% oxazolone solution (4-ethoxymethylene-2-phenyl-2-oxazolin-5-one, Sigma-Aldrich, 300 mg oxazolone in 7.5 ml 96% ethanol and 2.5 ml acetone) applied on their shaved breast and abdomen. Subsequently, 4 days after sensitization, ear thickness was measured under general anesthesia (isoflurane/N_2_O/O_2_) and thereafter all animals were challenged with 25 µl 0.8% oxazolone solution per ear (32 mg oxazolone in 3 ml 96% ethanol and 1 ml acetone). Once more, 24 h after the challenge, ear swelling was measured under general anesthesia (isoflurane/N_2_O/O_2_) to determine the Th1 immune response. At day 20 after tumor inoculation, body composition, i.e., fat mass (FM), lean body mass (LBM), bone mineral density (BMD) and bone mineral content (BMC), of the animals was determined under general anesthesia (isoflurane/N_2_O/O_2_) by densitometry using a PIXImus imager (GE Lunar, Madison, WI, USA). Subsequently, mice were euthanized under anesthesia by cardiac puncture, blood was collected and sampled in heparin tubes. Skeletal muscles m. extensor digitorum longus (mEDL), m. tibialis anterior (mTA), m. gastrocnemius (mG) and m. soleus (mS) from both hindlimbs were dissected and weighed and frozen at –80°C. Tumor, epidydimal fat, spleen, liver, kidneys, thymus, heart and lungs were dissected, weighted and frozen at –80°C. In tumor and mTA the phospholipid fatty acid content was assessed by GC, as previous described by Faber *et al.* [30].

### Statistical analysis

All data are expressed as mean ± standard error of the mean (SEM). Statistical analysis was performed using IBM SPSS Statistics (version 19; SPSS Inc. Chicago, IL). A mixed model ANOVA followed by LSD *post hoc* analysis was used to compare groups in *in vitro* experiments. Univariate ANOVA followed by LSD *post hoc* analysis was used to compare groups in the C26 animal experiment. Statistical significance was defined as *P* & 0.05.

## Abbreviations

BMD: bone mineral density
BMC: bone mineral content
DPA: docosapentaenoic acid
DHA: docosahexaenoic acid
EPA: eicosapentaenoic acid
FA: fatty acids
FBS: fetal bovine serum
PUFA: poly unsaturated fatty acids
TB-C: tumor-bearing control
TB-EPA: tumor-bearing EPA
TB-FO: tumor-bearing fish oil
TB-TO: tumor-bearing tuna oil
mTA: tibialis anterior muscle
